# RETRACTION: Long Non‐Coding RNA Sox2OT Promotes Coronary Microembolization‐Induced Myocardial Injury by Mediating Pyroptosis

**DOI:** 10.1002/ehf2.15242

**Published:** 2025-02-04

**RETRACTION:** L. Xuan, D. Fu, D. Zhen, D. Bai, L. Yu, and G. Gong. “Long Non‐Coding RNA Sox2OT Promotes Coronary Microembolization‐Induced Myocardial Injury by Mediating Pyroptosis,” *ESC Heart Failure*, 9, no. 3 (2022): 1689‐1702. https://doi.org/10.1002/ehf2.13814.

The above article, published online on 18 March 2022 in Wiley Online Library (wileyonlinelibrary.com), has been retracted by agreement between the journal Editor‐in‐Chief, Dr. Pitor Ponikowski; The Heart Failure Association of the European Society of Cardiology; and John Wiley & Sons Ltd. The retraction has been agreed following a report by the authors that confirmed Figure 5D in their article had been published previously in articles by other authors. The authors responded to an inquiry by the publisher and stated that this figure was provided by an external laboratory, but did not supply original data and images. Because the duplicated figures each reported on different treatments of cells, the data reported in this article is considered unreliable and the journal must issue a retraction. The authors did not respond to our notice regarding the retraction.

